# Innovating Occupational Safety Training: A Scoping Review on Digital Games and Possible Applications in Agriculture

**DOI:** 10.3390/ijerph18041868

**Published:** 2021-02-15

**Authors:** Lucia Vigoroso, Federica Caffaro, Margherita Micheletti Cremasco, Eugenio Cavallo

**Affiliations:** 1 Institute of Sciences and Technologies for Sustainable Energy and Mobility (STEMS), National Research Council of Italy (CNR), Strada delle Cacce, 73, 10135 Torino, Italy; lucia.vigoroso@stems.cnr.it (L.V.); eugenio.cavallo@stems.cnr.it (E.C.); 2 Department of Education, University of Roma Tre, via del Castro Pretorio 20, 00185 Rome, Italy; 3 Department of Life Sciences and Systems Biology, University of Turin, via Accademia Albertina, 13, 10123 Torino, Italy; margherita.micheletti@unito.it

**Keywords:** agriculture, gamification, human–work system interaction, safety, serious game, training engagement, e-learning

## Abstract

Digital games have been successfully applied in different working sectors as an occupational safety training method, but with a very limited application in agriculture. In agriculture and other productive sectors, unintentional injuries tend to occur with similar dynamics. A literature review was carried out to understand how occupational risks are addressed during game-based safety training in different productive sectors and how this can be transferred to agriculture. Literature about “serious game” and “gamification” as safety training methods was searched in WEB OF SCIENCE, SCOPUS, PUBMED and PsycINFO databases. In the forty-two publications retained, the computer was identified as the most adopted game support, whereas “points”, “levels”, “challenges” and “discovery” were the preferred game mechanics. Moreover, an association can be detected between the game mechanics and the elements developed in the game. Finally, during the game assessment, much positive feedback was collected and the games proved to be able to increase the operators’ skills and safety knowledge. In light of the results, insights are provided to develop an effective, satisfying and engaging safety game training for workers employed in agriculture. Games can be best used to learn and they are certain to improve over the next few years.

## 1. Introduction

In several working environments, workers are exposed to on-site hazards which can result in fatalities and serious injuries [1]. Despite improvements in working conditions, the high number of unintentional injuries reported in different industrial and productive working sectors continues to represent a relevant issue [2]. Thus, the promotion of safety behaviors and the need to increase the employees’ risk perception in the workplace has become one of the primary focuses [3,4]. Previous studies [2,5] proved that adequate safety training promotes workers’ involvement in safe behaviors both at individual and group level, reduces employees’ perception of work stress, and increases safety commitment and injury prevention [2]. In addition, in the 1990s, the US Occupational Safety and Health Administration (OSHA), recognized that these training programs need to be embedded in a broader safety system structure, including the employer commitment, hazard analysis or surveillance, hazard control and prevention [5].

To deliver safety and health training, several methods are used [6,7] and they can be more or less passive. The least engaging methods include lecture, adoption of videos and written materials as pamphlets, whereas progressively more engaging training methods consist of feedback interventions, computer-based instructions associated with hands-on demonstrations, and finally behavioral simulation as the most engaging training method [8]. As stated by [8], a more engaging training method allows greater knowledge acquisition and more transfer of training to the work setting, thereby improving behavioral safety performance and reducing negative safety and health outcomes. Unfortunately, highly-engaging safety training methods such as hands-on demonstrations are considered rather costly compared with other less engaging safety training methods. Thus, despite its low level of engagement, the most common safety training method is represented by lectures [9].

More recently, new forms of occupational safety training are emerging and different approaches have been implemented [1,3] especially with the aim to make safety training less passive and more engaging. Indeed, thanks to the use of technology, training has become more flexible in terms of time management, and it is a cost-effective alternative to practice, since it can provide realistic and effective simulations of real-life experiences [10]. In particular, digital games are creating a more exciting, engaging and interactive learning experience [11], enhancing the process of learning in terms of practice. A game as a training method can provide the players, and in this case the employees, an adequate hands-on learning opportunity by means of simulation. Following the principle of “learning by doing” [12], workers can understand and discover what they have to do and which behaviors to avoid, by experiencing themselves [13]. Game-based interventions have the advantages to focus players’ attention on specific tasks and they can be used when it is prohibitively expensive or simply too dangerous to allow trainees to use the real equipment in the real world [14]. In such situations, they will spend time learning valuable lessons in a “safe” virtual environment yet living a close-to-life-like experience. Often the convenience is to permit mistakes during training for a safety-critical system.

Several simulation tools and software applications identified with the name of serious games or gamification tools have been developed [14,15] and can be differently defined. In brief, serious games represent real-world processes and events with the purpose of solving a problem, but they are more similar to classic video games; whereas gamification tool is a more recent method and refers to applying game thinking, and elements to a non-traditionally ludic context [16], providing also rewards and incentives in forms of points, badges and virtual goods to increase players’ motivation to find effective problem-solving strategies [14,16]. Moreover, a game system is made up of specific frameworks of game design, that are game mechanics and dynamics: game mechanics represent the main elements of the game and have the power to guide the player action (e.g., points, levels, discovery, etc.), whereas game dynamics can be described as players’ interaction with mechanics and the satisfaction of their desires (e.g., achievements, competitions, rewards, etc.) [17].

Previous studies showed that serious games and gamified learning methods have been particularly and successfully applied in different business fields [11,16] and in sectors where technologies and mobile applications are used, such as automotive manufacturing [18], occupational health settings including medicine, surgery and rehabilitation [19,20], sports (e.g., baseball, [21]) and in hazardous working sectors such as firefighting [22], construction [1] and mining [23].

### Safety Training in Agriculture and Aim of the Present Study

Agriculture is worldwide recognized as one of the most hazardous productive sectors [24,25]. The high risk of fatal or non-fatal injuries is due to the large variability of the tasks the operators have to perform, depending on crops, operations (seeding, weeding, harvesting, etc.), the machinery and tools adopted, the, sometimes extreme, weather and climate conditions they have to be carried out with, and the daily and seasonal exposure, as well as the lack of a strict standardization of the work in general [26,27,28]. Simple operations, such as mounting and dismounting a vehicle, depending on weather conditions, operator’s experience and behavior, represent a not negligible risk which may result in serious injuries [29,30].

Engaging workers in safety training is widely acknowledged to play a pivotal role in promoting occupational safe behaviors in the agricultural sector [31]. With regard to this, earlier research has proved that adopting visual tools and adding visual features to the training material is appreciated and can increase the level of workers’ engagement [31,32]. Nevertheless, safety training in agriculture still takes place using traditional and conventional methods such as lectures and classroom activities in which trainers use displays, pamphlets and posters to integrate their verbal explanation [9].

Agriculture does not seem to go at the same speed as other sectors in the development and application of new technologies [33], including innovative methods for workers’ safety training. The few studies available in the agricultural sector where digital games were introduced as a training method, refer to crop management and agroecology learning, teaching the operator how to use specific smart farming technologies and adopt innovative practices [34,35,36]. To our knowledge, even fewer studies focused on games as a safety training method: the studies conducted by [37,38] deal with agricultural machinery driving training and the need to motivate operators toward changing attitudes and thinking about safe behaviors. In detail, in the game proposed in [37], the player has to find clues and information to piece together the story related to an All-Terrain Vehicle (ATV, also known as Quad) injury in which a boy was involved, and understand the economic effects of this injury on his family and community. ATVs are frequently used to move over large farms and carry light loads. They are one of the major causes of fatalities and serious injuries in countries such as the USA, Australia and New Zealand [39]. In the study conducted by [38], a virtual environment was developed in which the player, through a simulation, could familiarize themself with agricultural machinery knowledge and basic operational techniques. The purpose of the game was to reduce the safety issues related to the agricultural machinery driving training.

However, the risks in agriculture are not only linked to driving activities, but to many other factors and the unintentional injuries that occur, share similar dynamics with other occupational sectors. A work system indeed includes “one or more workers and work equipment acting together to perform the system function, in the workspace, in the work environment, under the conditions imposed by the work tasks” [40] (p. 2) and each interaction between these components deserves attention since it may be a potential source of risk. In several workplaces, it is important to educate and train workers to pay attention to mechanical risks (injuries of cuts and/or crushing caused by machinery use), to manage dangerous situations and follow correct procedures in case of fire or presence of dangerous substances, to use adequate personal protective equipment (PPE) and finally, to educate workers to coordinate or manage rescue activities. Therefore, it is useful to understand how these risks are addressed and discussed during training where game-based interventions have been adopted in sectors other than agriculture, to understand how the difficulties and the obstacles during the training are solved.

Based on all these considerations, this scoping review aims at identifying and synthesizing the results currently available in the literature about the game-based safety training adopted in different occupational sectors, in terms of risks addressed, game mechanics and dynamics, and technological support used. Considering the potential provided by the use of digital games technologies, the analysis at first, will help to understand which risks in the interaction between the worker and the other components of the work system have been considered in previous gamified training interventions, and then which game mechanics, dynamics and technologies are able to offer a satisfactory and effective experience; subsequently, starting from these results, insights for future game-based safety training development for workers employed in agriculture will be discussed.

## 2. Materials and Methods

The literature search for the scoping review was carried out in December 2020. The relevant articles were searched for in four databases: WEB OF SCIENCE, SCOPUS, PUBMED and PsycINFO. Two parallel types of keyword searches were carried out: at first, the search terms “gamification” AND “safety” AND “training” OR “education” were used to identify relevant articles; subsequently, the search terms “serious game” AND “safety” AND “training” OR “education” were used. Publications from peer-reviewed journals, empirical studies and conceptual papers, were included. The review included articles reporting investigations conducted in any geographical area.

**Figure 1:**
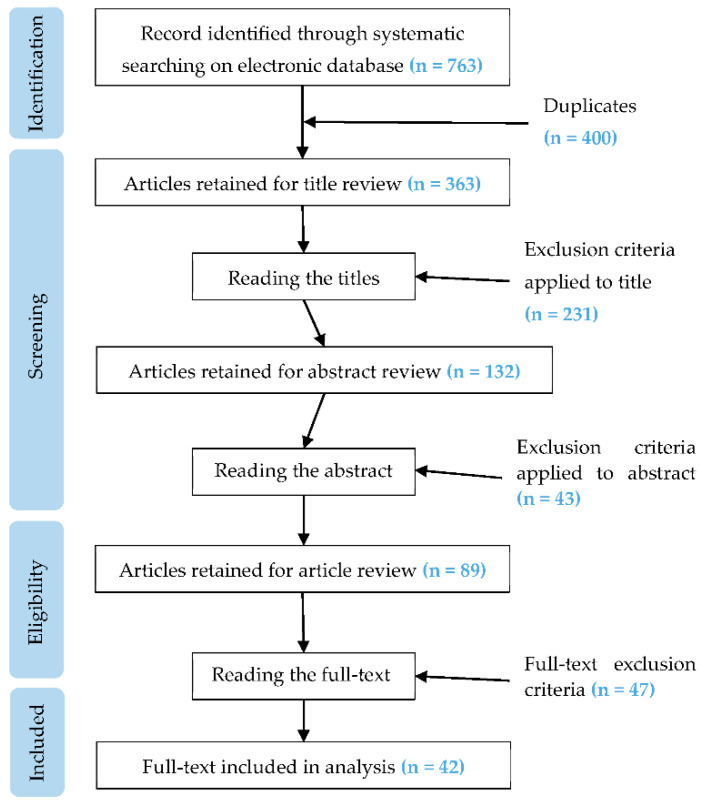
Flow diagram outlining the search and study selection process.

After duplicates were removed, the articles were screened in order of titles, abstracts and then full text. To be included in the literature review, the publications had to meet the following criteria: (i) publication examined a game-based training intervention; (ii) the topics of the game addressed issues related to risks in the interaction with machinery or equipment, work procedures, workplace environment and other workers, (iii) the study reported information on at least one of the following variables: users’ evaluation of the game developed or description of the game mechanics and dynamics used, (iv) the full-text available should be published in English. Figure 1 shows the articles selection process for the review.

The screening was performed independently by two authors, and any disagreement was discussed until unanimity was achieved. Once the papers eligible for inclusion were selected, for each study were recorded: the working sector in which the game was applied, the technology used, the sample involved, the game settings (in terms of mechanism, dynamics and number of players) and the findings. No quality assessment tool for publications or research considered by this study is used, since a scoping review does not aim at critical appraisal [41].

## 3. Results

A total of 763 articles containing the search terms were identified. After the removal of duplicates, the remaining 363 articles were screened on the basis of inclusion criteria applied to the titles, abstracts, and full text. At the end of the screening process, 42 publications were retained to be included in the final review analysis (Figure 1) to answer the research questions of the present review. The retained articles range from 2007 to 2020, with more than 50% in the last three years, demonstrating that the interest in the adoption of this type of training is rather recent.

Overall, it is possible to divide the analyzed studies into two main groups: the first one reporting the development and presentation of the games, and the second one related to game assessment. Almost all the studies provided a more or less detailed description of the game: some studies limited the description to the task required to the players, while others provide more details about the objects of interaction and even a wider explanation of the graphic interface adopted [23,42,43,44,45,46,47]. Regarding game assessment, nearly half of the studies evaluated the games in terms of usability, playability, satisfaction and players’ propensity to adopt games as a new safety training method [23,42,43,44,45,47,48,49,50,51,52,53,54,55,56,57,58,59,60,61,62]. Other forms of evaluation included training effectiveness through game performance (based on scores and player’s ability to complete levels) [37,47,48,52,58,63,64,65,66,67,68,69,70,71] or through the comparison between safety game training with traditional training (i.e., performed through lectures, videos and power-point presentations) [22,23,42,47,51,53,60,72].

In the following sections, we will describe the information reported by the retained studies regarding: the working sector in which studies are performed, participants involved in training interventions, risks addressed and similarities with agriculture, game frameworks (i.e., mechanics and dynamics), technology used to develop the game, and game satisfaction and effectiveness.

### 3.1. Working Sectors Considered and Participants Involved

Concerning the working sectors considered in the examined studies, they can be summarized as follows: industrial sector (including also automotive and construction sector) (n = 25), mining (n = 4), fire and firefighters (n = 3), aviation (n = 2), maritime sector (n = 1), business (n = 1) and other generic and not specified situations in which hazards can occur (e.g., restaurant businesses and offices and related services, n = 6). Regarding the number of participants involved in the testing of the developed game, 64.3% of the studies considered a sample with less than one hundred users (ranging from 3 to 97 participants), the 16.7% involved a sample with more than one hundred participants (ranging from 114 to 280 participants), while in the remaining 19% of the studies, the number of participants involved in the game testing was not specified. Among the thirty-three studies which described the sample size involved, it was also possible to observe that 50% of them involved only students or novices, 38.2% involved only professionals and experts, while 11.8% considered both types of users together. In studies involving novice users, the sample size ranged from 8 to 280 participants, while in studies with expert users, the sample ranged from 3 to 200 participants. Moreover, both novice workers and experts were involved when investigating safety in industrial sectors, mining, firefighters and maritime sector; in addition, experts and workers who hold managerial positions were considered in the aviation and restaurant business [56,57,68].

### 3.2. Technologies Used

In the selected studies, three main technologies were detected: computer games, virtual reality (VR) and augmented reality (AR) (which also includes mixed-reality—MR). Twenty-five studies adopted a computer game, that is a computer-controlled game where players interact with objects displayed on a screen; fourteen studies used VR, which consists of a simulated experience in which players, wearing goggles with a screen, are fully immersed in a 3D environment close to real life; only four studies used AR and MR, which merge the real and virtual life overlaying the real-world environment with virtual objects (see Table 1 to detect in which studies the virtual glasses were used to simulate the 3D environment).

### 3.3. Risks Considered and Game Frameworks

Table 1 reports for each study examined, the risks considered and the corresponding topics suggested for training in the agricultural sector by the International Convention and the International Labour Office (ILO) codes of practice [73,74], the game mechanics adopted and the number of players for whom the game training was designed and developed. In detail, some studies deal with more than one risk; whereas regarding the training topics classification in agriculture, they were subdivided into: general provision (in which the competent authority and the rights and duties of employers and workers are considered), personal protective equipment (PPE) adoption, machinery safety, ergonomics and handling of materials, chemicals and biological exposure and agricultural installation (which includes fall risk, fire risk and electrical risk). As it can be seen in Table 1, gamified training adopted in other occupational sectors addresses types of risks that are also relevant in agriculture.

With concern to the number of players involved, only six studies [44,54,56,68,75,76] consider developing the game also as a multiplayer platform, while the other studies refined the game only for single players. However, some studies [45,47,54,56,57,60,63,64,68,77] also included one or more non-player characters (NPCs), i.e., those characters in a game which are not controlled by the player but by the computer. NPCs characters can be more or less interactive with the player. In details, in six out of ten studies [47,60,63,64,68,77], the NPCs are not particularly interactive, they cannot start a relevant dialog for the game development purposes with the player and can be considered as a sort of decoration of the virtual environment, to make it seem “alive” or more realistic. Whereas, in the remaining four studies [45,54,56,57] the NPCs are a little more interactive, since they actively cooperate with the players: NPCs can perform tasks that are given to them [56] or provide messages and requests that must be solved in a timely manner [54,57] or may be directly influenced by the decisions made by the player [45]. In particular, in this last case, the NPCs can be injured, die or become increasingly stressed and irritated, and may choose to leave the workgroup. Decisions taken by players influence the NPCs’ behaviour.

At last, a recurrent association can be detected between the game mechanics and the elements developed in the game. In some studies, the development of the details of the surrounding environment is considered as an important additional element to be taken into consideration [47,51,54,68,77], i.e., different ground conditions, smoke or adverse weather that can limit the employer action in specific working conditions. In these studies, moving forward into the game scenes, the physical and environmental factors were evaluated as elements able to increase the difficulty of the game. Generally, when these elements are considered within the game, gameplay mechanics lean towards the “challenge” game mechanics.

Regarding the game mechanics used in the considered studies, some of them blended one or more mechanics. Based on this, 52.4% of studies were based on “Points”, 33.4% studies developed the game using “Levels”, 57.1% of studies were based on “Challenge”, 40.5% studies used “Discovery” and only 4.8% of the studies implemented a mechanic named “Virtual good and space” (see Table 2 for the definition of these mechanics).

During game development, also the playtime appeared to have a pivotal role in increasing the player’s motivation and engagement. With reference to “time”, many studies specified that the game provides real-time feedback and interaction, so that a specific action corresponded to an immediate reaction and vice versa, to ensure more realism of the game (e.g., [43]). Instead, when considering effective playtime, only eleven out of forty-two studies have highlighted this aspect. Even though in some studies a countless game repetition was allowed to ensure that users’ training was complete [45,65], the necessary time to complete the game depended on the number of levels and tasks required. In these studies, the game training was designed to be played and tested for a duration ranging from three minutes [64] up to a maximum of one hour [47,62]. In particular, in [68] two missions were requested, the first one needed to be completed in eight minutes and the second one in 20 min. The same time was reported also in [79] and [61]. On the other hand, a time of 12 min was estimated in [23], 15 min in [58,65], 30 min in [65] and 45 min were reported in [45].

### 3.4. Assessment of Game Satisfaction and Effectiveness

The studies reporting a game training assessment can be divided into three groups: (i) studies assessing game usability, playability or satisfaction (n = 21), (ii) studies analyzing game effectiveness through the game performance (n = 14) and (iii) studies investigating the effectiveness of the developed safety game comparing it with the traditional training methods (i.e., lectures, videos and power-point presentations) (n = 9).

Regarding the first group of studies, much positive feedback was collected in several studies. Overall, game training was defined as interesting, intuitive, enjoyable, able to offer a funny learning experience and to hold the users’ attention [23,43,44,45,47,49,51,54]. Game training was particularly appreciated when “lots of options” and “realistic aspects” were provided [43,44,45,56]. In addition, in the studies in which participants were asked if they were interested in playing the game again, favorable responses have always been given [45,54]. Among the whole studies retained, only in [47] the “language” issue was raised. Indeed, the following sentence is reported, “One student with English as his second language commented that the game supported his learning better than the lecture as the game contained only a few texts and allowed him to repeat and experiment” (p. 8).

However, some negative feedback was also encountered. For instance, in [47] the proposed game was evaluated as too easy and with too many occupational safety and health controls in it; in [49] the game was considered boring due to its repetitive tasks, while in [44,61] the hand gestures needed to use the AR glasses and difficulties on the VR interface and the navigation control scheme were a common problem detected by many participants. In particular, with regard to the VR technology in [23] the operators “mentioned that playing such a VR game is very tiring, and adapting to dizziness is a big challenge” (p. 118647). In addition, in [49,53], the participants involved and employed in construction and mining sectors respectively, reported negative evaluations concerning the absence of tactile sensation, force feedback and feelings of isolation.

In the second group of studies identified, training effectiveness was evaluated analyzing participants’ knowledge of safety issues after the game, observing the scores achieved by the participants and their capability to detect, remove and/or avoid the hazards introduced in the simulated game environment [52,64,66]. Some other studies in this group tested the increase in skills and safety knowledge using a pre–post-test, before and after the game training session [37,65,67]. Overall, nearly all studies reported high levels of comprehension and high levels of performance for the tasks required in the game, demonstrating that the game can be an effective training alternative. In contrast, in [66] the majority of the operators involved in the game test provoked the loss of their Avatar’s life since they did not identify and removed the hazards.

Finally, the last group of studies included the smallest number of studies, in which a control group (trained with traditional methods or devices) was compared with an experimental group (trained with a game). Participants who received game training performed a statistically significantly better hazard perception compared with the in-class lecture group [42,72], video-media group [22,23] and those who read the users’ manual [51]. Moreover, in [23] a follow-up test was proposed, demonstrating that VR training allows users to maintain longer memories of safety issues compared with video media.

## 4. Discussion

In the last few decades, digital games have rapidly grown and become a solution to enhance human motivation in various areas, including occupational training [1,16]. Indeed, from the present review, it emerges that literature has begun to mention the terms “gamification” and “serious games” in occupational sectors from 2007 and, afterward, a higher number of studies have been successfully conducted, especially in the last three years. Considering the still rare use of digital games for safety training in the agricultural sector, the present study investigated how occupational risks and hazards are addressed in gamified training interventions in other productive sectors, to identify possible applications in agriculture. The literature review showed that gamification has been applied to train workers on all the main types of risks and avoidance behaviors that may also be encountered in agriculture, in the interaction with equipment and machinery, environment, work procedures and other workers. Here, we will discuss the advantages and disadvantages of some game characteristics reported in the analyzed studies. Overall, the capability of engaging users and the potentiality that this type of technology can offer was undoubtedly highlighted in many studies [1,47,84]. Indeed, in [1] computer game training is recognized as being able to overcome three main limitations compared with traditional methods: “limited representation of the actual workplace situations”, “limited consideration for workers who have low literacy” and “failure in maintaining trainees’ attention” (p. 109). Indeed, in the previous research in which game training was compared with traditional methods (i.e., videos or power-point presentations), the digital games (using both computers or other devices) resulted to be more engaging and the participants reported higher levels of knowledge after having received the training [53], and higher levels of satisfaction. However, despite the game-training being able to recreate the working environments and the worker’s decision-making processes, in some occupational sectors such as mining, the players may be satisfied with their gameplay performance, but they also may complain about the impossibility of interacting with real materials [49,53]. In addition, although participants reported stronger emotional responses when comparing the VR technology with the computer-desktop display, some of the retained studies reported that dizziness restricted the time of the game tests, representing one of the main factors that hinder the popularity of VR technology [23].

When considering other factors limiting the adoption of game technologies as a training method, it should be taken into account that different technologies may not have the same costs [85]. The studies here analyzed did not expressly mention the related costs, however, in the case of virtual and augmented reality technologies, the adoption of high-performance 3D projectors and running a cluster of computers requires high costs of purchasing and service, which cannot be borne by all types of organizations/institutions [85]. Thus, the game platform and the respective technology must be chosen based on the availability and possibility of the training organization.

Regarding the game frameworks, the designers applied a limited number of game mechanics and interface elements. Overall, the game mechanics included, by and large, points, challenges and levels. Future projects could use more complex game mechanics solutions, and a wide variety of interface elements and rewards can be mixed [84]. Moreover, it can be further investigated how the environmental features can affect and/or improve the playability and the game mechanics. Additional features such as environmental details and NPCs, allowed to keep the training session more enjoyable and immersive [86]. However, in the retained studies, these features were explored only in the research conducted by [45]. Indeed, the design of non-player characters, specific weather conditions and addition of physical elements (e.g., smoke) that are perceived as authentic by the players is a critical success factor in the development of an engaging educational game [56,87], especially when a multiplayer game version is not yet available. Providing the player with interactive choices, the use of symbols and/or dialogue and interactions with NPCs [88] can establish an emotional link between the player, the other characters and the environment [45,87]. Based on this, we hypothesize also that a nonlinear-gameplay adoption, in which the game story proceeds following the player’s choices or player’s success or failure at a specific challenge and the possibility of multiple endings, can positively increase the dramatic effect and the attention of players [45,89]. Furthermore, NPCs may be used to simulate the mental pressure exerted by supervisors on workers, being able to experience these kinds of situations within a video game may have significant changes in social relationships between farm operators [90]. 

Concerning the agricultural sector, even though many activities are carried out by the operators alone, some other activities require the operators’ cooperation [26], exposing them to the risk of injuries. Therefore, it could be interesting to provide specific details of the surrounding environment and develop more NPCs to discuss and successfully complete more complex tasks with them.

To create a digital game it is necessary to satisfy some fundamental requirements that can influence human behavior and motivation [17,91]. In light of the results discussed, some insights are provided to develop an effective, satisfying and engaging safety-game training for workers employed in agriculture:

- even though different technological supports (computer, VR and AR) have proved to be effective and satisfactory [22,23,45,47,56], the computer game technology can be detected as the most used and “practicable” one both in terms of ease of use and play rules (e.g., the game could be downloaded and can be played on one’s own personal computer) and in terms of costs;
- the game may allow the players to look around to become aware of their environment and has to be able to recreate the decision making processes that operators encounter in different hazardous situations, with the aim to improve operators’ capability to react to hazards in real situations [43,54,75];
- games should be developed with multiple levels and should be structured with different and increasing levels of difficulty to be more appreciated [47,51,68];
- the game scene needs to be well contextualized within a suitably designed work environment allowing the player to identify specific hazards [43,44,45,71];
- simulated characters (NPCs) should be present since they motivate trainees by being credible, trusting and helpful [37,45,46,56,60];
- the game should have simple gameplay with few core activities and a limited set of core game mechanics, but with some variations in tasks, since introducing new little elements allows to enhance the challenge and sustains the motivation during the game [92];
- the game could be based on rewards to increase players’ engagement [50,79];
- the game should give the player the possibility to practice their skills, through a “trial and error” approach, and ultimately win, to increase motivation and engagement [55];
- players must be allowed to retry as many times as necessary to complete the game; however, the total playing time for the computer-game must not exceed one hour, in order not to be boring or frustrating [62,65].

The insights provided here are particularly relevant for the agricultural training context, since gamification has been under-investigated as a training method in agriculture.

However, these insights can also be extended and tested in other hazardous sectors in which serious games and gamification have already been used as safety training methods. We acknowledge that the insights we provided for the agricultural sector are only a starting point, and future studies may be developed to adapt different game mechanics to specific work activities, farm characteristics and operations in specific environmental conditions (e.g., adverse climatic and weather conditions, presence of slopes or different kinds of terrain).

Regarding the limitations of the analyzed studies, the issue concerning the sample involved in the game assessment has be to mentioned. Many previous studies were based on small samples (less than ten users) (e.g., [57,64]), but a number of researchers have pointed out that these samples are not enough to ensure the validity of the results [1,93]. Therefore, in future research, it is recommended to consider the involvement of larger samples. Moreover, some research questions for future studies in agriculture but also in the other sectors considered, can be identified based on the reviewed literature. In particular, which is the role played by individual variability in terms of gender, age, nationality, and level of working experience (i.e., novice or expert) when assessing game usability and/or satisfaction and/or effectiveness? Indeed, previous studies showed that all these variables can affect attitudes toward technology adoption and how people interact with technologies.

Concerning the gender issue, in the last decade, different trends have been reported for males and females regarding their interest in using computer games, preferred game genre [94] and attitudes towards innovative technologies, including e-learning [42,95]. Considering the increased number of female video game players (forty-two percent of all game players) [42,96] and the increasing feminization of agriculture [97] it would be interesting to investigate how people of different genders interact with game-based safety training, their preferences and performances are.

Furthermore, the age-related changes in cognition can affect the requirements of interface design [98], making it relevant for future studies to involve both young and older users’ in the development and assessment of game-based training. This aspect deserves particular attention also when addressing the farming population since, like other sectors, agriculture is experiencing progressive ageing of the working population [99].

Serious games have proven to be able to help learners rapidly acquire basic communication skills in foreign languages and cultures [1,100]. However, people from different countries may react in different ways to a set of stimuli or rules, and teaching methods [95,101]. For instance, countries with a higher power distance are reluctant to change in problem-solving situations, preferring conventional learning methods and structured learning situations [95,100]. Therefore, analysing the effect of different cultural context when developing and evaluating serious games as a training method, would represent a key challenge. Finally, in a game usability test, novices and experts may have different interests in exploring the virtual environment [102]. The studies included in this review, which involved both novices and experts in game assessment, did not perform a separate analysis for each group. However, [102] showed that novices tended to be keen on technology and spent some time playing with the devices before actually starting the design review and assessment tasks. In contrast, professional experts seemed to be more focused and jumped right into the activity. Different behaviors toward the game can influence the effective playtime and the player’s perception of the game itself, therefore future research on game-based safety training should take the user’s level of experience into account.

As detected in the present review, previous studies have already demonstrated the superiority of game-based training over the traditional methods in terms of effectiveness (such as knowledge acquisition) and satisfaction [23,42,51,53]. However, considering the growing interest in this innovative training and the existence of different technologies that can be used for such purposes, future studies may perform a comparison between these technologies. For instance, previous literature on gaming confirms that no particular differences can be found in players’ performance and game usability between immersive and non-immersive games [103]. Whereas, in contrast, other results reported that better performance was achieved using computer-desktop digital games rather than VR methods [104,105]. It would be relevant to understand whether certain types of skills can be better acquired by adopting a simple computer game or by using a more complex augmented reality or virtual reality equipment.

Finally, none of the retained studies reported effectiveness data in terms of transferability to on-job real performance. This issue should be addressed in future research to ensure that game-based training can facilitate effective skills transferability [106].

Some limitations of the present review should be mentioned. First, the search was limited to English language publications. Second, unpublished studies, with a limited distribution (the so-called “grey” literature [107]) were not included in the review because they were not validated by a peer-review process and indexed in bibliographic databases. Finally, even though it was performed by two authors independently, the screening of the studies was characterized by a certain degree of subjectivity. This was particularly important when deciding how to categorize some game mechanics and dynamics.

Regarding the insights provided for the development of game-based training in agriculture, we are aware that training can solve many safety issues, helping workers to recognize hazards and risks in the workplace and to avoid them. However, there are other sources of stress in agriculture–as working alone for farmers [108], or pressure from supervisors in the case of farmworkers [109], pressure from governmental regulations, financial and management issues and lack of control [74,110], poor safety attitudes—that can contribute to unintentional injuries occurrence and should be tackled not only through training but through targeted multi-level interventions.

## 5. Conclusions

This scoping review allowed us to identify the existing serious games and gamified solutions applied to safety training in different occupational sectors and to propose some possible developments and adaptations to the agricultural sector. Creating engaging game-based safety training methods is particularly relevant in agriculture, considering its high hazardousness. Based on the results of the present review, gamified safety training in agriculture may be developed starting from previous experiences in other sectors, since gamification has been adopted to address risks and behaviors which correspond to safety needs in agriculture. The present review showed that digital games can represent an effective and satisfying alternative solution to hands-on demonstrations, but some studies have also revealed the weakness of digital games linked to the absence of some sensory aspects such as haptics, which is particularly difficult to recreate in virtual simulations. Despite this weak point, in today’s scenario, learning methods have become increasingly digitalized and e-learning, in particular, is becoming much more appreciated for its flexibility, availability without space and time constraints and cost-effectiveness. In particular, based on the dynamics developed during the COVID-19 pandemic period, the use of digital platforms has doubled and prove to be an effective instrument to train workers. The general perception of the usefulness of games to support learning will certainly improve over the next few years; we believe that research should no longer focus on whether games may be used for learning, but instead should investigate how games can be best used for learning.

## Figures and Tables

**Table 1:** Hazards encountered in the articles reviewed and their counterparts in the agricultural sector and the relationship between the game system and the risks discussed in the retained studies.

Source	Risk Considered	Parallels with Training Topics in the Agricultural Sector	Description	Game Mechanics Used	Players
Albert et al., 2014 ^2^ [67]	Chemical risk; Falling risk	Chemicals and biological exposure; Agriculturalinstallation	Within different working environments, the players had to identify the relevant hazard sources. The game provided feedback in case of correct or wrong answers.	Points; Discovery	single player
All et al., 2017 [72]	Fire risk	Agricultural installation	The game was based on three small interactive minigames in which the player could earn coins for taking the correct steps (e.g., answering a multiple choice question on what is the correct action to take), performing the right action (e.g., alarm internally by calling the correct number) or performing the correct order of actions (e.g., steps to activate a fire extinguisher).	Points; Levels; Challenge	single player
Alshiar et al., 2019 ^2^ [61]	PPE adoption; Use of machinery; Falling risk	PPE; Machinery safety; Agricultural installation	The construction site consisted of the following 3D objects: building, workers, vehicles, hole in the ground and interactable objects. When the player selected an interactable object, a menu with two buttons, “unsafe” and “cancel,” appeared. If the player clicked the “unsafe” button, then the following actions occured: (1) The object was marked unsafe permanently; (2) The object became non-interactable; (3) A new menu appeared with possible reasons why the selected object is unsafe; (4) The player could hide the menu and continue examining the scene.	Challenge; Discovery	single player
Amal et al., 2016 [55]	Use of machinery	Machinery safety	Operations with the crane machine were divided into different phases: 1. understanding crane components using a 3D model that can be rotated 360 degrees on display, 2. crane driving simulation, 3. lifting and dropping operations, and 4. final assessment.	Challenge	single player
Brown et al., 2018 [45]	Fire risk; Falling risk	Agricultural installation	The player had the role of a foreman with the aim to evacuate his crew in the face of a rapidly unfolding mine disaster. Hazards as roof falls, flammable gases, expiring and defective respirators, inoperative refuge chambers, electrical faults and broken escape lines were subsequently added.	Challenge	single player
Bükrü et al., 2020 ^2^ [66]	General; PPE adoption; Use of machinery	Agricultural installation; PPE; Machinery safety	The player had to identify the common hazards in outdoor construction work environments. The scene contained many 3D objects to make it more life-like. Some typical sounds and noise levels of an angle grinder appeared, unless the player started wearing virtual ear muffs to block out noise and protect his/her hearing. The main task for the user is to identify the hazards common in outdoor construction work environments.	Discovery	single player
Burt et al., 2018 [70]	Driving vehicle	Machinery safety	The player made decisions that allowed them to navigate in the game while controlling a forklift within the building. On each of the five levels, the player had to collect an item and move it to a container but encounter different hazards.	Points; Challenge; Discovery	single player
Cai et al., 2017 ^2^ [77]	Fire risk; Driving vehicle	Agricultural installation; Machinery safety	The player could control the cranes from the control cab. The lifting operation included boom extending, luffing, and rotating, as well as load hoisting. During the lifting operation, the player had to pay attention to the riggers who assisted the moving of the loads at the beginning or at the end of the lifting task, helping to keep the load at a safe distance from the surrounding with the use of suitable ropes.	Challenge	single player
Catton et al., 2018 [78]	Use of machinery; Electrical risk	Machinery and safety; Agricultural installation	The game was based on four steps: hazard identification, risk assessment, establishment of control measures and performance of a review for current efficacy.	Points; Levels; Challenge; Discovery	n.a ^1^
Chodan et al., 2017 [79]	Management	General provision	Different challenges could be played: classical multiple-choice, true or false or games like finding problems and finding the safe paths. After the player has completed the game, he is moved to the game result page.	Points; Challenge; Discovery	single player
Dawood et al., 2013 ^2^ [52]	Chemical risk; Falling risk	Chemicals and biological exposure; Agricultural installation	A brief overview of what the session was about and details on logging into the environment were provided. Then, using a worksheet the players could record what types of hazards were present during the exploration of the working environment.	Points; Discovery	single player
Dawood et al., 2014 [71]	Chemical risk; Falling risk	Chemicals and Biological exposure; Agricultural installation	Adopting a worksheet, the players could record the hazards that were present in the working environment during its exploration.	Points; Discovery	single player
Dholakiya et al., 2018 [80]	Chemical risk; PPE adoption	Chemicals and biological exposure; PPE	The players learned: 1. the basic rules of laboratory safety, i.e., the importance of PPE, storage and handling dangerous chemicals; 2. the hazardous consequences in case of mistakes; 3. to analyze unintentional injuries using safety engineering theories.	Points; Levels	n.a ^1^
Dickinson et al., 2011 [44]	Use of machinery	Machinery safety	Three tasks were required: 1. To safely retrieve the toolbox from the bottom of a trench; 2. After the partial trench collapse, the player must investigate the main reasons for the collapse; 3. The player was responsible for the planning of five trenches on the job site, each with its own soil types, obstacles and shoring requirements.	Levels; Challenge	single player with peer suggestion
Din et al., 2019 [42]	Electrical risk; Falling risk	Agricultural installation	The player had to recognize the hazard depicted in various scenarios from the options displayed on the screen. Scenarios were developed taking into account: site location and access, material storage options, housekeeping, pedestrian safety, overcrowding, trenching and excavation safety, formwork erection and removal decisions, use of PPE, laying underground utilities, parapet adequacy for fall protection, fragile roofing (skylights, corrugated fiberglass), material storage and overhead power lines, excavation and underground power lines.	Points; Levels; Discovery	single player
Eller et al., 2018 ^2^ [75]	Fire risk	Agricultural installation	Developed as a collaborative virtual environment. It was focused on extinguishing fires and navigating the building. Players could interact with different objects or communicate with other participants. A tracker is attached to a real fire extinguisher that is accurately represented also in the virtual environment.	Points	single and multi-player option
Gilotta et al., 2018 [81]	Use of machinery; PPE adoption	Machinery and safety; PPE	The player had to recognize different operational steps: 1. ergonomics (check avatar PPE, incongruous postures), 2. documents and procedures, 3. equipment and workstation, and 4. operating procedures.	Points	n.a ^1^
Golovina et al., 2019 ^2^ [64]	Use of machinery; Electrical risk; Chemical risk	Machinery safety; Agricultural installation; Chemicals and biological exposure	The player employed in a construction site had to pick, walk, and finally place five recycling bags, one after the other, in a nearby container. During the game, the player interacted with the skid steer load and crane load	Challenge; Discovery	single player
Gonzales et al., 2009 [69]	Driving vehicle	Machinery safety	Drive task was performed under fatigue conditions and different types of roads, to understand how this condition reduced alertness and caused degrees of performance deterioration.	Challenge	single player
Greuter et al., 2013 [47]	Fire risk;PPE adoption; Management	Agricultural installation; PPE; General provision	The players had to recognise hazards on construction sites, and identify OHS communication and reporting processes. NPCs workers tried to accomplish tasks on the construction site, but they were hindered by hazards that prevented them from doing their work. NPCs were not able to manage the hazards by themselves and were injured if they came in contact with the hazard. To avoid these injuries, the player had to resolve the hazard using the OHS controls displayed in a menu at the bottom of the screen.	Points; Levels; Challenge; Discovery	single player
Hafsia et al., 2018 ^2^ [49]	PPE adoption	PPE	The player was a construction worker who had to carry out five different specific actions for the stabilization of the forestay; the players had also to pay attention to the place where a kickstand was placed to avoid any injury.	Discovery	single player
Hall et al., 2016 [58]	Fire risk; Management	Agricultural installation; General provision	The player learned to adjust any further decisions made in case of hazards, manipulated the environment with the aim to keep the NPCs safe.	Levels; Discovery	single player
Harper et al., 2018 ^2^ [50]	Use of machinery; PPE adoption	Machinery safety; PPE	During the game, a real grinder was used on a virtual block. To play, users have to wear the correct PPE, mark out the desired cut with a ruler and pen, cut the block using the grinder and remove the waste with a hammer.	Challenge	single player
Jiang et al., 2016 ^2^ [51]	Driving vehicle	Machinery safety	The player drove the tractor through different terrains such as sand, concrete road and pebbled way. Rain or heavy fog was added to simulate severe weather conditions, affecting the players’ visibility and increasing the difficulties of tractor driving.	Points; Challenge	single player
Kamkuimo et al., 2020 ^2^ [65]	Chemical risk; PPE adoption	Chemicals and biological exposure; PPE	The player had four missions: 1. identify situations in which someone is likely to inhale silica dust in the worksite; 2. resolve twelve situations associated with ways to eliminate the risk; 3. acquire individual and collective protective measures against silica dust in different situations; 4. become aware of the consequences of silicosis.	Levels	single player
Kuindersma et al., 2017 [57]	Driving vehicle	Machinery safety	The game allowed the players to have: 1. a better situation awareness, 2. workload management, 3. application of procedures 4. problem-solving and decision-making; The players interacted with non-player characters receiving messages and requests that must be solved in a timely manner to prevent catastrophic situations.	Points; Levels; Challenge	single player
Kwegyir-Afful et al., 2020 ^2^ [62]	Use of machinery	Machinery safety	By means of an immersive 3-D simulation, the task included removing the filter cover of the machinery, installing new filters and replacing the filter cover after the exercise.	Challenge	single player
Lanzotti et al., 2019 [48]	Biomechanical overload; PPE adoption	ergonomics and handling of materials; PPE	Two tasks were explained: 1. two correct postures for pulling and one for pushing were simulated. 2. the press workers operate in a virtual scene which included a station for PPE, crowbar hanger and the mould press machine.	Points; Challenge	single player
Leong et al., 2013 [82]	General	Agricultural installation	The player had to indicate the hazards in the scene as a preparation for the actual site visit assessment.	Points; Levels; Discovery	single player
Liang et al., 2019 ^2^ [23]	Falling risk	Agricultural installation	When scaling rocks, the players could identify hazards related to the randomly distributed loose rocks and hazards related to signs of unstable ground: 1. loose bolts, 2. loose material around bolt plates, 3. slabbing or crushing of ribs, 4. movement of fractures or slip faces, 5. bulges in the mesh, 6. cracks in shotcrete, 7. dry spots on roof or ribs and 8. loose material on the floor.	Points; Levels	single player
Lovreglio et al., 2020 ^2^ [22]	Fire risk	Agricultural installation	The game summarized the main steps of using fire extinguishers: pull, aim, squeeze and sweep. Four types of fire incidents were designed and the player performed the same actions in each of the four scenarios developed (i.e., warehouse, electrical, office and worksite).	Challenge	single player
Mallet et al., 2008 ^2^ [54]	PPE adoption	PPE	The game allowed the players to move through the mine. To complete the tasks, the player counted cross-cuts, go through many doors, and found belt crossovers. Two main locations must be find, but the second one was more difficult to find. The non-player characters encountered provided information and further directions.	Challenge	single and multiplayer option
Matsas et al., 2015 ^2^ [43]	Use of machinery	Machinery safety	The player has to interact with buttons, located on the workbench: the green one allowed to start the training and the red emergency one to stop the task. Pushing the button, a robot moved and handed the patch over to the player-avatar and the player had to approach the robotic arm paying attention not to collide with the robot’s body.	Challenge	single player
Mayer et al., 2013 [63]	Fire risk; Falling risk	Agricultural installation	The players were in charge of safety procedures at the drilling site and were required to fill out a hazard report. The players could also interact with non-player characters through a scripted dialogue text menu. Hazards ranged from simple safety procedures (maintenance) to more complicated, simultaneous and multiple procedures such as lifting and hoisting.	Points; Levels; Challenge; Discovery	single player
Pietrafesa et al., 2020 [76]	General; Management	Agricultural installation; general provision	The focus was on the conceptual equation between safety and business growth; the game included progressive incentives and prizes, but whenever a worker suffered an injury the player loses points and positions in the high-score table.	Points; Challenge	single-player and multiplayer
Proctor et al., 2007 [68]	Driving vehicle	Machinery safety	The player flew a simulated two flight plans: 1. from the home station to a remote location in mountainous terrain to rescue a down crew; 2. after picking up them, the player flew to an away airport experiencing noticeable air turbulence and considering the additional weight of the passengers.	Challenge	multiplayer option
Rapp et al., 2019 [56]	Management	General provision	The game aimed to: 1. address declarative knowledge, 2. foster skills, and 3. raising awareness and changing attitudes towards a positive understanding of health and safety promotion in the workplace. Four phases characterized the game: 1. the players plan the current calendar week, 2. the players play a daily working routine and earn money, 3. the players have to react to a “critical incident” that takes place and 4. the players get feedback for their actions.	Levels; Challenge	single and multi-player
Regent et al., 2013 [46]	Electrical risk	Agricultural installation	The players collected clues by interviewing “witnesses” or consulting documents to compile a list of facts relating unintentional injuries which mirror real causes of electrical risks. There were four investigations: 1. maintenance and repair of a meter, 2. repair of a medium voltage overhead network, 3. repair of a low voltage underground cable and 4. lock-out in a substation. Using the information collected, the players, guided by the trainer, analysed the unintentional injuries.	Points; Discovery; Virtual goods and space	single player
Somerkoski et al., 2020 ^2^ [60]	Fire risk;	Agricultural installation	The fire alarm was triggered on the lower floor; The player would not see any sign of smoke until s/he had left the room and reached one of the lowest two floors. The game contained fifteen tasks such as reacting to the fire alarm, taking the right exit door, making an emergency call and avoiding areas with smoke. The correct decision increased the score.	Points	single player
Stothard et al., 2010 [53]	Falling risk	Agricultural installation	Five simulator scenarios relating to “Working at Heights” were presented. The scenarios included an elevated work platform task, a ladders task, an excavation task and two scaffolding tasks. Task complexity varied between scenarios.	Discovery	single player
Torres et al., 2020 ^2^ [59]	General	Agricultural installation	The study objective was to compare the participants’ perceptions of usability and engagement between the interactive and non-interactive versions of the video.	Challenge	single player
Wang et al., 2020 [83]	Use of machinery	Machinery safety	The players learned how to assemble machines, learned about the assembly process and got to know the parameters of different basic components such as screws, motors, bearing.	Points; Levels	single player

^1^ n.a = information is not explicit or understandable from the paper drafting. ^2^ Studies in which a 3D viewer was used.

**Table 2:** Game mechanics used in the retained studies and their corresponding game dynamics. Some studies are described with more than one game mechanic.

Game Mechanics	N. of Studies in Which It Was Used	Corresponding Game Dynamics	Game Dynamics Description
Points	22	Reward	Help in achieving the primary desire of being rewarded
Levels	14	Status	Motivation for the people to improve player status by achieving a level up
Challenge (and quest)	24	Achievement	When the player likes to accomplish difficult challenges
Discovery	17	Pattern recognition	Players feel intrinsically rewarded just for having discovered hidden elements.
Virtual good and space	2	Self-expression	Enable the users to create a virtual identity that can be used for self-expression
